# Vitamin D, Essential Minerals, and Toxic Elements: Exploring Interactions between Nutrients and Toxicants in Clinical Medicine

**DOI:** 10.1155/2015/318595

**Published:** 2015-07-29

**Authors:** Gerry K. Schwalfenberg, Stephen J. Genuis

**Affiliations:** ^1^Department of Family Medicine, University of Alberta, No. 301, 9509-156 Street, Edmonton, AB, Canada T5P 4J5; ^2^Department of Medicine, University of Calgary, 2935-66 Street, Edmonton, AB, Canada T6K 4C1

## Abstract

In clinical medicine, increasing attention is being directed towards the important areas of nutritional biochemistry and toxicant bioaccumulation as they relate to human health and chronic disease. Optimal nutritional status, including healthy levels of vitamin D and essential minerals, is requisite for proper physiological function; conversely, accrual of toxic elements has the potential to impair normal physiology. It is evident that vitamin D intake can facilitate the absorption and assimilation of essential inorganic elements (such as calcium, magnesium, copper, zinc, iron, and selenium) but also the uptake of toxic elements (such as lead, arsenic, aluminum, cobalt, and strontium). Furthermore, sufficiency of essential minerals appears to resist the uptake of toxic metals. This paper explores the literature to determine a suitable clinical approach with regard to vitamin D and essential mineral intake to achieve optimal biological function and to avoid harm in order to prevent and overcome illness. It appears preferable to secure essential mineral status in conjunction with adequate vitamin D, as intake of vitamin D in the absence of mineral sufficiency may result in facilitation of toxic element absorption with potential adverse clinical outcomes.

## 1. Introduction

The medical literature has achieved general consensus that vitamin D levels throughout much of the world, as reflected by population measurements of 25(OH)D_3_ levels, are inadequate [1]. About 2/3 of the population in northern climates are considered deficient with average 25(OH)D_3_ levels of 67 nmol/L [2], much below the 100–150 nmol/L level that has recently been associated with preferred health [3]. There are many papers emphasizing the benefits of supplemental vitamin D in order to achieve levels that are protective for many diseases [4, 5]. There has been recent concern expressed, however, that consumption of excessive doses of supplemental vitamin D may pose certain risks and potentially confer harm to individuals. With recognition that vitamin D intake can also facilitate the uptake of toxic elements, the objective of this review paper is to explore known interactions between vitamin D, essential minerals, and toxic elements in order to provide clinical recommendations regarding the supplemental use of this important vitamin.

This review was prepared by assessing available medical and scientific literature from Medline, as well as by reviewing several books, nutrition and toxicology journals, conference proceedings, government publications, and environmental health periodicals. A primary observation, however, was that limited scientific literature is available on the issue of vitamin D in relation to essential and toxic elements. The format of a traditional integrated review was chosen as such reviews play a pivotal role in scientific research and professional practice in medical issues with limited primary study and uncharted clinical territory [6].

## 2. Vitamin D Adequacy and Safety

As vitamin D acts epigenetically in the regulation of over 2700 different genes by acting on vitamin D responsive elements [7], it is not surprising that considerable literature confirms the necessity of achieving adequate 25(OH)D_3_ in order to attain optimal health. A recent article suggests that 25(OH)D_3_ levels >30 nmol/L have significantly lower all-cause mortality than levels <30 nmol/L [8]. Levels above 78 nmol/L are considered beneficial for bone health and maintaining normal parathyroid hormone [9]. Cancer prophylaxis may not be fully realized until levels are over 90 nmol/L [10]. Benefits in countering infections such as tuberculosis and influenza may require levels of over 100 nmol/L [11] and levels at or above 120 are associated with the lowest mortality [3].

While there is abundant evidence confirming potential harms associated with deficient vitamin D, as well as much research displaying the enormous benefits of supplementation to replete and maintain adequate vitamin D indices [38], uncertainty has arisen regarding levels that are considered too high. A recent article, for example, showed increased 90-day mortality rates in hospitalized patients with preadmission levels of 25(OH)D_3_ <50 nmol/L or >150 nmol/L [3]. Such findings have raised concern that levels of 25(OH)D_3_ greater than 150 nmol/L may not be optimal. This U shaped phenomenon of benefit only within a specific range and risk outside of this range has been suggested in other articles as well. The risk for pancreatic cancer, for example, allegedly increases at higher vitamin D levels [39] but on further analysis this finding may have been a statistical artifact due to the chosen cut-off point groupings [40].

The risk of potential harms associated with higher levels, however, is dismissed by others with the contention that 25(OH)D_3_ levels of 225 nmol/L can be achieved with ordinary sunlight and are thus considered normal. Furthermore, levels of <375 nmol/L have been shown in some research to not result in any evident toxicity [41]. In review, there is insufficient study of supplementary doses of vitamin D which result in 25(OH)D_3_ levels higher than 150 nmol/L to make firm conclusions. Just the same, there has been preliminary exploration of pathophysiological mechanisms that might account for potential risks associated with higher 25(OH)D levels.

## 3. Vitamin D and Inorganic Elements

One point of note is that adequate 25(OH)D_3_ is associated with improved absorption of essential elements including calcium, magnesium, iron, phosphate, zinc, and copper [12]. What has largely been forgotten, however, is that higher levels of 25(OH)D_3_ have been linked to enhanced absorption of toxic elements such as aluminum, cadmium, cobalt, and lead as well as radioactive isotopes including cesium and radioactive strontium [12]. It has also been observed in the chick that vitamin D increases zinc and cadmium absorption [42]. Vitamin D has no effect on mercury absorption in the chick intestine but increases cobalt and iron absorption in the presence of low calcium [43]. In children, elevated 25(OH)D_3_ levels in the summertime are associated with a seasonal increase in blood lead levels via increased intestinal absorption [44]. It is also well recognized that bioaccumulation of such toxic metals in turn appears to disrupt physiological functioning of vitamin D within the body. For example, accrual of lead or cadmium diminishes the activity of vitamin D, by blocking the normal renal synthesis of active 1,25-dihydroxyvitamin D [12]. There is also evidence discussed in the literature of myriad adverse effects that various toxic metals including cadmium, lead, mercury, and aluminum can have on normal biological processes including uptake, absorption, and assimilation of assorted essential minerals [16, 17]—which may consequently result in health problems. Toxic metals themselves can also accrue in various tissues and have been directly linked to various adverse health outcomes [45–47].  Table 1 provides an overview of the complex interaction between vitamin D and various inorganic elements—both required minerals and toxic metals.

The question therefore arises as to whether the alleged rise in morbidity and mortality associated with elevation of 25(OH)D_3_ (>150 nmol/L) may be, in part, associated with the increased accumulation of toxic metals—a common concern in contemporary society [48]. To the authors' knowledge, however, no studies have been done to date which measure accrued levels of toxic metals in population groups in direct relation to 25(OH)D_3_ levels. One of the challenges with the assessment of this hypothesis is that much of the reported biomonitoring of toxic elements in population groups has been confined to unprovoked blood or urine levels of toxicants—which often underestimate the body burden. Most toxic elements and compounds tend to sequester in tissues and may not be evident on blood or urine testing [49]. Lead, for example, may be abundant in bone and brain where it tends to accumulate, with potentially little evidence of such accrual with blood or urine testing [50].

It is also evident that vitamin D does not act solely in isolation. Impaired vitamin D functioning and insufficient levels of essential minerals can have synergistic and cumulative adverse action on biological function with significant pathophysiological impact. For example, vitamin D metabolism is dependent on sufficient magnesium as a cofactor for vitamin D to bind to its transport protein and for this vitamin to convert into the active form via hydroxylation in renal and hepatic sites [51]. Furthermore magnesium deficiency may upregulate the 24(OH)ase enzyme in the kidney resulting in catabolism of active vitamin D [51]. Insufficiency of magnesium has been associated with many adverse clinical effects including depression [52], anxiety [53], and cardiac problems [54] and has recently been found to be associated with impaired immune function [55] and to inversely affect C-reactive protein [56]. It is estimated that more than 68% of US adults are consuming levels of magnesium below the recommended daily allowance (RDA) [57]. Factors that may enhance magnesium deficiency, states such as accrued toxic metals possibly resulting from elevated vitamin D in the absence of sufficient minerals, may thus have an impact on metabolic function.

Furthermore, any determinant such as accrued toxic metals that would exacerbate zinc deficiency also has a potential detrimental impact on physiological function. Along with iron, boron, manganese, and copper, the essential mineral zinc is important as a cofactor in bone health. Specifically, zinc facilitates bone formation by stimulating the osteoblast [58]. While the average daily intake of zinc is considered to be only 46–63% of the RDA, various toxic metals have a detrimental impact on zinc uptake into the body (Table 1). Additionally a study on mineral content of foods has found that more than 80% of Americans do not achieve the RDA or the estimated safe and adequate daily dietary intake of calcium, magnesium, copper, zinc, and iron. The result of such widespread deficiency may be increased risk of toxic element absorption [59].

The complex interaction between the essential element calcium, vitamin D, and toxic metals is also evident in various reports from the literature [Table 1]. While no more than 800 mg of calcium a day may be required when vitamin D levels are adequate, the typical diet in North America may be inadequate to supply even this limited amount [60]. Furthermore, as is noted in Table 1, toxic metals may impair calcium uptake resulting in deficiency states. While much recent attention has been devoted to the finding that excess calcium intake may actually cause harm, increasing the risk of myocardial infarction by 31% and stroke by 20% [61], it is important to remember that sufficiency of calcium is required for normal physiological function, a clinical state that may be compromised by vitamin D insufficiency or toxic metal bioaccumulation.

## 4. Vitamin D Supplementation

Adequate sun exposure in warmer climates or consumption of vitamin D containing foods such as fatty seafood in northern areas has traditionally been the preferred means to achieve adequate vitamin D status. However, higher latitudes experience ultraviolet B sunlight intensities that are too weak for extended periods to induce sufficient vitamin D skin synthesis. Furthermore, insufficient consumption of vitamin D containing foods frequently occurs because of dietary preference, or avoidance because of concern about toxicant accrual in foods such as mercury in fish. As a result, vitamin D supplementation is being encouraged from many sources as a means to secure adequate intake in order to maintain optimal biological functioning.

With adequate sunlight and food consumption, it appears that there are natural mechanisms to secure preferred vitamin D levels and to prevent excessive bioaccumulation. With sun exposure, for example, enzyme downregulation appears to occur as higher levels are achieved so that diminished vitamin D skin production, absorption, and assimilation occur [62]. This inherent protective approach, however, may not be evident with supplemental intake of isolated vitamin D ingestion. With supplementation particularly for populations living in more northern latitudes, how does one secure optimal vitamin D levels in clinical settings without exceeding healthy levels?

Just as one might measure specific indices such as hemoglobin or potassium levels in patients inclined to be low in these biochemical markers, monitoring of individual 25(OH)D levels in clinical settings is the preferred way to secure an optimal vitamin D status in individual patients. As there is variation in response to vitamin D supplementation as a result of factors such as weight and toxicant levels that influence uptake and absorption of vitamin D, measurement is the only way to confirm optimal vitamin D status, to ensure compliance with instructions, and to preclude excessive or dangerous levels. While there have been many studies that confirm the benefits of vitamin D supplementation in specific groups [4, 5], there has been a paucity of studies that actually measure individual levels in population groups after a specific level of supplementation.

The varied response to specific levels of vitamin D supplementation is evident in one such study—a nursing home study supplementing with 2,000 IU daily for more than 5 months (Table 2). The residential population with an average age of 80.7 (*N* = 68) achieved an average 25(OH)D level of 119.3 nmol/L with this level of vitamin D ingestion [63]. Further analysis of this data reveals that 12 patients or 22% achieved levels less than 100 nmol/L but that 6 patients or 9% reached levels of >150 nmol/L. At this level of supplementation, about 6% of patients would not achieve levels considered necessary for good bone health at 78 nmol/L but only 78% would have levels between 100 and 150 nmol/L. It appears that about 4000 IU of vitamin D might be required to allow a significant portion of the population to achieve the desired 100–150 nmol/L. With this level of supplementation, none of the participants would reach a commonly accepted dangerous 25(OH)D level of >375 nmol/L.

In another study (Table 2) of the general population (*N* = 1430) at a northern latitude [2], projections were made based on average responses to specific levels of vitamin D supplementation. In this report, only 22% of the 1430 patients were found to have levels between 100 and 150 nmol/L. Within the 1% of patients found to have levels over 150 nmol/L of 25(OH)D_3_, more than 73% admitted to pronounced levels of sun exposure, regular artificial sun tanning at tanning studios, or both. The highest level recorded was 216 nmol/L in a patient that both had sun exposure and was sun tanning, Once again, none of the participants reached levels anywhere near or >375 nmol/L.

In addition, a recent risk assessment for vitamin D toxicity with supplemental doses found no evidence of toxicity using 10,000 IU daily for a six-month period [64]. As a result of the evident safety of using considerable supplemental doses of vitamin D, the Institute of Medicine (IOM) has recently raised the maximum allowable amount of vitamin D to 4000 IU daily with no required monitoring for toxicity [65]. With variation in response to specific doses of supplemental vitamin D, monitoring of 25(OH)D levels with required dose adjustments appears to be the most effective means to secure adequacy and to preclude excessive levels.

## 5. Clinical Implications

There has been much debate in the medical literature about the preferred level of 25(OH)D, the optimal level of supplementation, and the degree of intake or levels that might be dangerous. In the medical literature as a whole, many researchers suggest that measured levels of 25(OH)D should ideally remain within the 100 and 150 nmol/L range [66]. This view is endorsed by the Vitamin D Society as lower levels are associated with inferior human health outcomes and higher levels might have the possibility of increasing risk of morbidity and mortality. As mentioned, some recent information suggests that vitamin D intake to achieve a minimum level of 120 nmol/L is associated with the lowest mortality [3], a recommendation that has been adopted by groups such as the “Vitamin D Council” and “Vitamin D Society.” A recent Endocrine Society recommendation suggests targeting for a 25(OH)D level value greater than 75 nmol/L. In order to ensure that individuals “true” 25(OH)D is greater than 75 nmol/L, they suggest aiming for a value of 100 nmol/L, a level that is not associated with toxicity [67].

Conversely, however, some prominent medical groups have differed in their recommendations. While the IOM (Institute of Medicine) agrees that 4,000 IU of vitamin daily is allowable and nontoxic, the actual recommended daily dose by this group is 600 IU daily [65]. This IOM recommendation has been put into question [68] as a significant statistical error has been identified in the way the recommendation was arrived at [69]. Furthermore, the IOM recommendations have been refuted by a study suggesting that it may take as much as 8800 IU of vitamin D daily to bring 97.5% of the population to levels of 50 nmol/L [69].

Because of practical concerns such as expense associated with testing, nonetheless, some have suggested that there is no point determining and following 25(OH)D measurements, with the rationale that most individuals are low and should simply be taking regular vitamin D supplementation. But the degree of supplementation will vary based on geographic area, degree of sun exposure, nature of the diet, level of toxicants, and so on. Annual testing has long been suggested for this reason [5]. Accordingly, while it is increasingly suggested that a certain range of 25(OH)D may be associated with preferred health outcomes, there may be huge differences in the required intake of supplemental vitamin D to achieve a specific 25(OH)D endpoint. For example, populations at higher latitudes would require significantly more supplemental vitamin D in order to achieve levels above 100 nmol/L compared to those living in warm sunny climates. Accordingly, annual biomonitoring of 25(OH)D levels is suggested when possible as the health benefits and resultant cost savings should far outweigh the expense of annual testing. The savings in healthcare dollars have been estimated to be in the range of 14 billion dollars in Canada [70], 187 billion in Western Europe [71], and 56 billion in the United States [72]. Essentially, it is estimated that the cost of biomonitoring would be about 5% of the cost savings.

Sufficiency of vitamin D has implications for other essential nutrients as this important vitamin is recognized to interact and maintain physiological function in concert with other vitamins and minerals. As discussed, absorption of essential minerals and toxic metals are all increased with more vitamin D, and insufficient levels of various essential minerals appear to facilitate toxic metal absorption [Table 1]. The majority of Americans, however, receive insufficient magnesium [73] largely due to the processing of foods where magnesium levels are reduced by as much as 400% [74]. Evidence suggests that intake of other essential minerals is also inadequate in many situations, resulting in a higher risk of toxic metal absorption. Hospitalized patients, for example, are prone to mineral deficiencies, particularly in the intensive care units [75]. Accordingly, in order to achieve an optimal vitamin D status and to minimize the risk of toxic element accumulation, securing intake of essential minerals through foods or supplementation in addition to adequate vitamin D levels is fundamental to achieving optimal health outcomes.

## 6. Conclusion

Several clinical recommendations are in order based on the presented information from the literature. Population studies across the world report low levels of vitamin D. Lifestyle changes and adequate supplementation are required to achieve optimal 25(OH) levels—thought to be about 100–150 nmol/L. From the studies listed in Table 2, it is evident that, in the average population in a country such as Canada with little natural UVB stimulation for >6 months of the year, only 22% of the population achieve levels to confer all the benefits (bone and nonbone) of vitamin D adequacy. Likewise supplementing with 2000 IU would achieve adequate levels in less than about 78% of the population. Blood monitoring is recommended on a yearly basis with sufficient supplementation to secure optimal levels (25(OH)D levels >100 nmol/L) as outlined above [5]. Such an approach would realize enormous savings of healthcare resources across the world.

It is important to recognize that vitamin D does not work alone but requires essential minerals to achieve its full benefit. Deficiency of minerals including magnesium, calcium, zinc, and iron is very common as outlined above. Recognizing the synergistic action of mineral deficiency with elevated vitamin D levels on the uptake of toxic elements, adequate intake of minerals needs to be ensured.

It is possible that the concern associated with excessive vitamin D might be explained by the increased absorption and bioaccumulation of toxic elements. Further study is required to explore this emerging concern. Just the same, efforts to reduce exposure to and accrual of toxic elements such as the diminution of emissions of toxic elements by industry are also indicated. This would reduce contamination by toxic elements in the air we breathe as well as deposition in soil and uptake into consumed foods, thus diminishing the risk of exposure and uptake of toxic metals, regardless of levels of vitamin D and essential minerals.

Finally, there is preliminary evidence that higher morbidity and mortality may be associated with excessively elevated vitamin D levels. This problem may be exacerbated by a deficiency of essential minerals, potentially resulting from inadequate dietary intake or the result of accumulated toxic elements. Therefore, efforts to secure mineral adequacy and to avoid toxic metal exposure and avoidance of potentially excessive vitamin D intake are suggested.

## Figures and Tables

**Table 1:** Interactions of vitamin D, essential minerals, and toxic elements.

Interaction	Vitamin D (VTD)	Calcium (Ca)	Magnesium (Mg)	Zinc (Zn)	Copper (Cu)	Iron (Fe)	Selenium (Se)
Vitamin D	NIL	↑ absorption of Ca [12]	↑ absorption of Mg [12]	↑ absorption of Zn [12]	↑ absorption of Cu	↑ absorption of Fe [12]	↑ absorption of Se

Cadmium (Cd)	↑ absorption of Cd [12] ↑ absorption results in ↓ active VTD (renal)	Low Ca intake results in ↑ Cd absorption and results in Cd osteodystrophy [13]	Low Mg intake = ↑ Cd absorption ↑ osteodystrophy	Cd competes with Zn for absorption replaces Zn on metallothionein [13]	↑ Cd decreases Cu absorption and interferes with Cu metabolism; increased Cu protects from Cd toxicity [13]	Cd decreases Fe absorption; low Fe intake = ↑ Cd absorption [14]	Se protects against Cd toxicity [15]

Lead (Pb)	↑ absorption of Pb [12] ↑ absorption results in ↓ active VTD (renal) and promotes Pb toxicity	Low Ca results in ↑ Pb absorption and ↑ Pb in tissues and brain to impair cognition; calcium and phosphorous supplementation decreases Pb absorption and retention [16, 17]	Increased calcium and magnesium may protect against lead induced hypertension in pregnancy [18]	Pb competes with Zn for intestinal absorption and replaces zinc on hem enzyme; Zn supplementation decreases tissue Pb accumulation [16, 17, 19]	Copper insufficiency leads to increased toxicity of Pb; dietary copper reduced Pb absorption [19]; together, iron and copper completely inhibited the effects of Pb	Low Fe intake = ↑ Pb absorption competing for transport system; supplementation may decrease Pb absorption and toxicity [17]	Se is useful as an adjunct in chelation treatment in Pb intoxication

Mercury (Hg)	No effect on absorption of Hg [43] Vitamin D may help detoxify the brain from excess Hg [20]	↑ Hg releases intracellular Ca stores disrupting neuronal transport Ca protects against mercury toxicity [21]	Mg protects against Hg toxicities but less than Ca [21]	Zn is protective against methylmercury damage [22]	Cu protects against Hg toxicities but less than Mg [21]	Iron protects against Hg toxicity; Hg exposure may result in iron deficiency [23]	Se protects best against Hg toxicity and binds mercury [15, 17]

Cobalt (Co)	↑ absorption of Co	N/A	N/A	Administration of Co increases Zn concentration in liver	Administration of Co increases urinary Cu excretion [24]	High iron interferes with Co absorption [25]	Cobalt may reduce the absorption of Se [26]

Aluminum (Al)	↑ absorption of Al [12]	Low calcium in presence of Al results in ↑ Al absorption and osteodystrophy [17]	Ca deficiency and low Mg intake result in ↑ Al absorption and Al induced neurodegeneration	Al may have a protective effect on testis in Zn deficiency state (rat study) [27]	Al may have a protective effect on testis in Cu deficiency state [27]	Low Fe intake = ↑ Al absorption	Se may have a protective effect from Al [28]

Strontium (Sr)	↑ absorption of Sr [12]	↓ intestinal absorption of calcium (competitive) must have adequate VTD present [29]	↓ intestinal absorption of Ca and Mg; Sr bone benefits disappear with low Mg [30]	Bone benefits disappear with low Zn [30]	Sr may reduce the level of Cu in the blood [31]	Sr competes for iron absorption	N/A

Arsenic (As)	Unknown	Ca has protective effects against As toxicity [32]	Mg may have protective effects against As toxicity [32]	Zinc may increase As elimination; mechanism is unknown [33, 34]	As may increase copper deposition in the kidney [35]	Iron is used as a precipitant to remove arsenic from water; the combination may cause hepatic damage in humans [36]	↓ or ↑ moderate Se will ↓ As toxicity [32] High level of Se may ↑ As toxicity [37]

NIL = no interaction, N/A = information not available, ↑ = increase, and ↓ = decrease.

**Table 2:** Vitamin D levels achieved in 2 studies done at northern latitudes.

	Number	Percentage
(1) Higher latitude statistics for high levels of 25(OH)D_3_, *N* = 1430 [2]		
Number of patients with >150 nmol/L of 25(OH)D3	15	1%
Number of patients with >100 nmol/L of 25(OH)D3	315	22%
Number of patients with ideal levels 100–150 nmol/L	300	21%
(2) Nursing home study using 2000 IU daily of vitamin D_3_ for >5 months, *N* = 68 [63]		
Number of patients with >150 nmol/L of 25(OH)D3	6	9%
Number of patients with >100 nmol/L of 25(OH)D3	54	78%
Number of patients with ideal levels 100–150 nmol/L	48	71%

^*∗*^All levels achieved in these patients were well below 375 nmol/L where side effects have been reported.

## Key Points

- Vitamin D sufficiency as reflected by a 25(OH)D level of about 100–150 nmol/L may be an optimal clinical endpoint, although controversy remains over the ideal level. Taking into account the vast number of population studies, in both temperate and equatorial climates, population levels are quite low in many jurisdictions and supplementation is recommended to achieve optimal levels.
- The IOM daily recommendation of 600 IU of vitamin D3 for adults <70 years old would not achieve these preferred levels in more than 50% of people and thus the IOM recommendation has been called into question as outlined above.
- Sufficiency of essential minerals is necessary to prevent bioaccumulation of toxic elements and to enhance activation of vitamin D related proteins.
- Excessive vitamin D intake may have detrimental effects, perhaps by enhancing the absorption of toxic elements.
- Levels of vitamin D supplementation required to reach optimal endpoints will vary depending on myriad factors and thus a single recommended dose for all may not be an optimal approach to secure vitamin D adequacy.
